# Influence of Thyroglobulin Autoantibodies on Thyroglobulin Levels Measured by Different Methodologies: IMA, LC-MS/MS, and RIA

**DOI:** 10.1210/clinem/dgae286

**Published:** 2024-04-30

**Authors:** Ivana Petrovic, Jonathan LoPresti, Shireen Fatemi, Andrew Gianoukakis, Kenneth Burman, Cristiane J Gomez-Lima, Caroline T Nguyen, Carole Ann Spencer

**Affiliations:** Division of Endocrinology, Diabetes, & Metabolism, Department of Medicine, Keck School of Medicine, University of Southern California, Los Angeles, CA 90033, USA; Division of Endocrinology, Diabetes, & Metabolism, Department of Medicine, Keck School of Medicine, University of Southern California, Los Angeles, CA 90033, USA; Department of Endocrinology, Kaiser Permanente, Panorama City, CA 91402, USA; Division of Endocrinology, Lundquist Institute, Torrance, CA 90509, USA; Division of Endocrinology, Diabetes and Metabolism, Harbor-UCLA Medical Center, Torrance, CA 90509, USA; Endocrine Section, Medstar Washington Hospital Center, Washington, DC 20010, USA; Endocrine Section, Medstar Washington Hospital Center, Washington, DC 20010, USA; Division of Endocrinology, Diabetes, & Metabolism, Department of Medicine, Keck School of Medicine, University of Southern California, Los Angeles, CA 90033, USA; Division of Endocrinology, Diabetes, & Metabolism, Department of Medicine, Keck School of Medicine, University of Southern California, Los Angeles, CA 90033, USA

**Keywords:** differentiated thyroid cancer, thyroglobulin antibodies, thyroglobulin measurement

## Abstract

**Context:**

Serum thyroglobulin (Tg) measured by immunometric assay (IMA) is prone to underestimation due to Tg autoantibody (TgAb) interference, often prompting reflex Tg measurement by liquid chromatography/tandem mass spectrometry (MS) or radioimmunoassay (RIA).

**Objective:**

IMA, MS, and RIA methodologies were used to measure serum Tg in TgAb-negative (TgAb−) and TgAb-positive (TgAb+) patients with either distant metastatic differentiated thyroid cancer (DTC) or hyperthyroidism (HY)—patients in whom a detectable serum Tg would be expected.

**Results:**

*When TgAb was absent*, all methodologies detected Tg in the sera of all DTC and HY patients and reported appropriate Tg trends and treatment responses for DTC patients with progressive distant metastatic disease, albeit with high between-method variability (> 30% coefficient of variability). *When TgAb was present*, all methodologies reported lower serum Tg levels for both DTC and HY groups vs their respective TgAb− group. No Tg was detected by IMA or MS in ∼50% TgAb+ DTC patients (6% had no Tg detected by RIA). Surprisingly, 5% of TgAb+ HY patients also had no Tg detected by IMA or MS. The inverse log TgAb/log Tg correlations seen for the TgAb+ HY patient group with all methods suggested the presence of a TgAb-associated serum Tg-lowering effect.

**Conclusion:**

(i) Between-method Tg variability necessitates method continuity when monitoring the Tg trends of TgAb− DTC patients. (ii) The presence and concentration of TgAb appeared to have a lowering effect on serum Tg measured by all methodologies (IMA, MS, and RIA). (iii) Since the reliability of Tg measured in the presence of TgAb is often uncertain, the TgAb trend (measured by the same method) may be a useful surrogate DTC tumor marker.

Thyroglobulin (Tg), the large (∼660 kDa) glycoprotein thyroid hormone precursor, is uniquely synthesized by thyroid follicular cells and co-secreted with thyroid hormones in proportion to the thyroid tissue mass (∼1 µg/L Tg per gram) (1, 2). The tissue-specific origin of Tg has prompted the use of serum Tg as a postoperative tumor marker for patients with treated differentiated thyroid cancer (DTC) (3, 4). Three methodologies (immunometric assay [IMA], mass spectrometry [MS] and radioimmunoassay [RIA]) are currently available for Tg measurement (5, 6). Sensitive automated IMA methods that use proprietary monoclonal antibodies (MAbs) to detect conformational epitopes on the dimeric Tg protein (7-9) have mostly replaced the older competitive RIA methods that employ polyclonal antibodies which detect a wider range of epitopes than MAbs (10-12). The MS methods that have become available use trypsin digestion to release a Tg-specific peptide that is immunocaptured and quantified by liquid chromatography–tandem mass spectrometry (LC-MS/MS) (6, 13-16).

Most thyroid tumors synthesize and secrete Tg protein, although the efficiency of neoplastic Tg secretion can be abnormal (17, 18). Furthermore, there can be molecular heterogeneity and conformational abnormalities in neoplastic Tg that can result from differences in mRNA splicing (19) glycosylation (20-22) and/or iodine content (23-25). Such abnormalities in the tertiary Tg protein structure have the potential to disrupt the conformational epitope targets of both IMA and RIA assays (2, 7) causing between-method differences (2, 26-29). In contrast, polymorphisms and post-translational modifications have the potential to produce false-negative MS results (30).

Interference caused by the Tg autoantibody (TgAb) which is present in up to ∼25% of DTC patients (31, 32) has been a persistent problem limiting the clinical utility of Tg measurement (6, 33) and is not reliably detected by a Tg recovery test (3, 31, 34). TgAb interference can cause either falsely low or high Tg values, depending on the methodology used, the TgAb concentration and patient-specific TgAb epitope specificity (35). The unidirectional (underestimation) TgAb interference typical with IMA methodology likely results from TgAb blocking the specific epitope(s) targets of the MAb reagents (1, 7, 36). TgAb can also interfere with RIA methodology causing either falsely high or low values, depending on the affinity and specificity of the polyclonal antibody and the characteristics of the ^125^I-Tg tracer (37). Although Tg MS methods were developed with the goal of overcoming TgAb interference (6, 13-16), Tg MS clinical sensitivity remains questionable given many reports that paradoxically undetectable MS-measured Tg levels are present in up to 20% to 50% of TgAb+ patients with structural DTC disease (6, 33, 38-40).

The persistent problem of TgAb interference (6, 33, 38) has prompted guidelines to mandate TgAb screening before serum Tg measurement is undertaken (3, 4). Some laboratories selectively reflex TgAb+ specimens to a non-IMA methodology considered more resistant to TgAb interference—usually MS or RIA (41). Serum TgAb levels tend to decline in response to successful treatment and rise with DTC progression (27). Since the reliability of Tg measurements made in the presence of TgAb is often uncertain, the TgAb trend is increasingly being used as a surrogate DTC tumor marker (3, 4, 42, 43), provided that TgAb is measured by the same method in preferably the same laboratory (3, 44). The goal of this study was to evaluate whether the presence of TgAb influences the clinical sensitivity of Tg measured by different methodologies (IMA, MS, or RIA) in patients in whom a detectable serum Tg would be expected as a result of DTC with distant metastatic disease (DMD) (3) or hyperthyroidism (HY) (45).

## Methods and Patient Groups

### Serum Specimens

Frozen archived sera from TgAb+ and TgAb− DTC patients with DMD or HY were retrieved from the University of Southern California Endocrine Laboratory and Harbor–University of California, Los Angeles Lundquist Institute repositories, respectively under institutional review board ethical approval for patient selection and the anonymization of the specimens for analysis. Both Tg and TgAb have been shown to remain stable in sera during long-term storage at −20 to −80 °C (35). The patients included in each of the 4 patient groups (DTC/DMD [TgAb+ and TgAb−] and HY [TgAb− and TgAb+]) were selected sequentially according to: (i) clinical status (DTC/DMD or HY); (ii) TgAb status (TgAb+ or TgAb−); and (iii) adequate specimen volume for testing. It took over 3 years to recruit the TgAb+ DTC/DMD patient group because only ∼25% of DTC patients have TgAb detected and only a minority (∼5%) of TgAb+ DTC patients have DMD (46). During this long specimen acquisition period, the sensitivity of the Tg MS methodology improved, prompting confirmatory remeasurement of sera without Tg detected by earlier less sensitive MS methodology.

### Fine Needle Aspiration Specimens

Three TgAb+ DTC patients with no Tg detected in their serum by IMA or Mayo Medical Laboratories MS (MS-M) had Tg measured in a fine needle aspiration (FNA) washout (FNAW) from a cytologically confirmed thyroid cancer lymph node metastasis. Each FNAW specimen was negative for TgAb, as would be expected from the high dilution employed by the procedure. The FNAW Tg was measured by IMA, MS-M, and RIA after dilution to a target Tg (10-20 µg/L) using a TgAb-negative/Tg-negative human serum pool.

### Tg Methods

Each method was standardized against the International Reference Tg Preparation CRM-457. The IMA and RIA methods had their functional sensitivity limits established according to guidelines (27, 47). MS test sensitivity was established using the less stringent limit of quantitation (LOQ) parameter (14-16)

#### Immunometric assay

The Tg IMA was the Beckman Access® immunochemiluminometric method (Beckman Coulter, Fullerton, CA, USA: [RRID:AB_131588]. This method had a second-generation functional sensitivity of 0.1 µg/L (27). Between-run precisions established for human sera measured over a 2-year period were 9.1%, 6.3%, 5.7%, 5.0%, and 5.0% at 0.17, 0.69, 7.8, 76, and 383 µg/L, respectively. High Tg values (> 450 µg/L) were diluted in serum diluent prior to measurement.

#### Radioimmunoassay

The Tg RIA was developed by the University of Southern California Endocrine Laboratory, Keck School of Medicine, University of Southern California, Los Angeles as previously described (8, 48) using a rabbit-antihuman first antibody (RRID:AB_3095735) and goat antirabbit second antibody (RRID:AB_3095888). The assay had a functional sensitivity of 0.5 µg/L. Between-run precisions established for human sera measured over a 2-year period were 14.9%, 7.8%, 8.5%, 7.9%, and 11.6% at 0.85, 4.0, 12.2, 18.6, and 33.9 µg/L, respectively. High Tg values (> 40 µg/L) were diluted in 14% bovine serum albumin.

#### Liquid chromatography–tandem mass spectrometry

We used trypsin to digest denatured serum proteins to produce a proteotypic Tg peptide that was immunocaptured before liquid chromatography–tandem mass spectrometry (LC-MS/MS) quantitation. The Mayo Medical Laboratories (MS-M) test detected the target peptide FSPDDSAGASALLR (6) with a sensitivity of 0.2 µg/L, whereas the Quest (MS-Q) test detected the target peptide VIFDANAPVAVR (14) with a sensitivity of 0.4 µg/L. All specimens had MS-M measurements, but specimen availability limited some MS-Q testing.

### TgAb Assay

The TgAb status of patients (positive or negative) was determined with the Kronus (RSR) semi-automated radioassay (Boise, Idaho, USA, aka RSR Cardiff, UK: [RRID:AB_3095085]), which uses ^125-^I-labeled Tg to bind TgAb in a diluted (1:21) serum specimen followed by precipitation of the TgAb-Tg complex by goat anti-human IgG. The assay had a functional sensitivity of 0.4 kIU/L (33). Between-run precisions established for human sera measured over an 18-month period were: 16.7%, 8.0%, 5.3%, and 9.3% at 0.8, 2.8, 7.7, and 14.2 kIU/L, respectively. High TgAb values (> 17 kIU/L) were diluted with serum diluent. Values ≥ 0.4 kIU/L were considered TgAb+ and values < 0.4 kIU/L were considered TgAb-negative.

### Thyroid Tests

Roche Elecsys methods were used to measure serum thyrotropin (TSH; RRID:AB_3095311), free thyroxine (FT4; RRID:AB_3095310) and free triiodothyronine (FT3; RRID:AB_2827368) in HY sera. The TSH method had a functional sensitivity of 0.01 mIU/L and between-run precision at 0.04, 0.5, 5.9 and 37 mIU/L of 6.3%, 2.6%, 2.3%, and 2.3% established over 18 months. The FT4 had an upper limit of 89 pmol/L and between-run precisions at 12, 33 and 63 pmol/L of 2.1%, 2.0%, and 3.7% established over 15 months. The FT3 had an upper limit of 49 pmol/L and between-run precisions at 3.1, 8.7 and 18 pmol/L of 3.7%, 2.7%, and 2.6% established over 15 months.

### Patient Groups

#### DTC patients with DMD

Of the DTC patients with DMD (n = 50; F/M ratio, 31:19), 19 were TgAb− (F/M ratio = 11:8) and 31 were TgAb+ (F/M ratio = 20:11). The site of metastatic disease was primarily lung (∼90% for both TgAb− and TgAb+ patients). Patient selection was weighted to favor TgAb+ patients in accordance with the study goals and the high TgAb positivity characteristic of hyperthyroidism (∼52%) (49). Median age at diagnosis for TgAb+ patients was 54 (range, 21-81) years, and for TgAb− patients was 57 (range, 19-81) years.

#### Patients with hyperthyroidism

The diagnosis of HY was based on clinical history and a suppressed TSH level. Of the HY patients (n = 66, F/M ratio, 50:16), 16 were TgAb− (F/M ratio = 13:3) with a median FT4 of 20 pmol/L (range, 12 to > 102 pmol/L) and median FT3 of 9 pmol/L (range, 5 to > 46 pmol/L); 50 patients were TgAb+ (F/M ratio = 37:13) with a median FT4 of 27 pmol/L (range, 9 to > 102 pmol/L) and median FT3 of 12 pmol/L (range, 5 to > 46 pmol/L).

### Statistical Analyses

Statistical analyses were performed using XLstat software together with Pearson correlations and a Spearman rank correlation coefficient calculations.

## Results

### Serum Tg in TgAb− and TgAb+ DTC/DMD Patients

**Figure 1.**
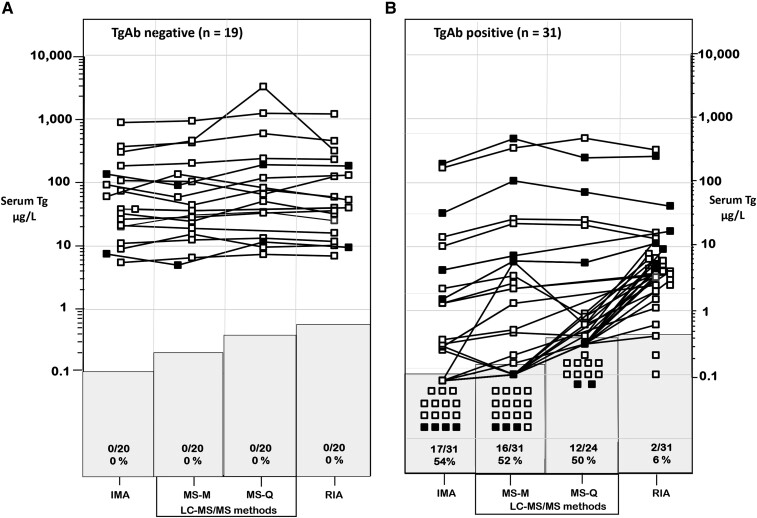
Serum Tg measurements made by different methodologies in patients with distant metastatic DTC who were either TgAb-negative (panel a) or TgAb-positive (panel b). Three Tg methodologies were compared: IMA, MS (MS-M = Mayo; MS-Q = Quest), and RIA. Patients with Tg below the assay sensitivity limit are indicated in the shaded areas and expressed as a percentage relative to the total number of tests performed with that method. Patients who died of DTC-related complications are shown by solid symbols.

In the absence of TgAb all patients had Tg detected by each method (Fig. 1a). However, large between-method numeric differences (median 30%; range, 8%-133% coefficient of variability [CV]), that exceeded the imprecision expected from using a single method (∼10% CV) were observed. Two patients died from disease-related complications (solid symbols).

In the presence of TgAb, Tg was detected in only ∼50% of patients by IMA or an MS test as compared with > 90% by RIA (Fig. 1b). However, Tg RIA measurements were lower for the TgAb+ vs TgAb− group (median 4.5 vs 36.3 µg/L, respectively, *P* < .01). Tg measured in a FNAW specimen from a metastatic lymph node of 1 TgAb+ DTC/DMD patient (with no Tg detected in serum by IMA or MS-M, RIA = 2.6 µg/L) was in the expected range for all methods (IMA: 24.0, MS-M: 24.9, and RIA: 22.5 µg/L, *P* > .1).

### Serum Tg Trends in 6 DTC/DMD Patients

**Figure 2.**
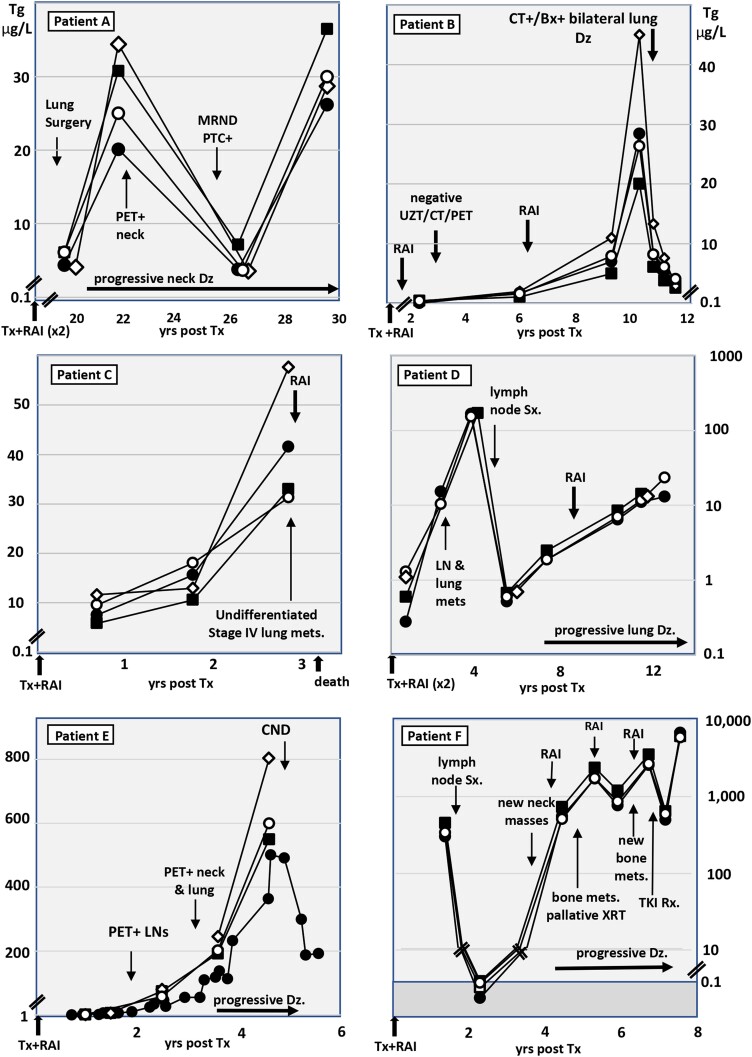
Serum Tg trends of 6 TgAb− patients treated for progressive distant metastatic DTC in the years following initial treatment with thyroidectomy (Tx) and radioiodine (RAI). The serum Tg was measured by different methodologies: IMA (solid circles), MS-M (solid squares), MS-Q (open diamonds), and RIA (open circles).

**Figure 3.**
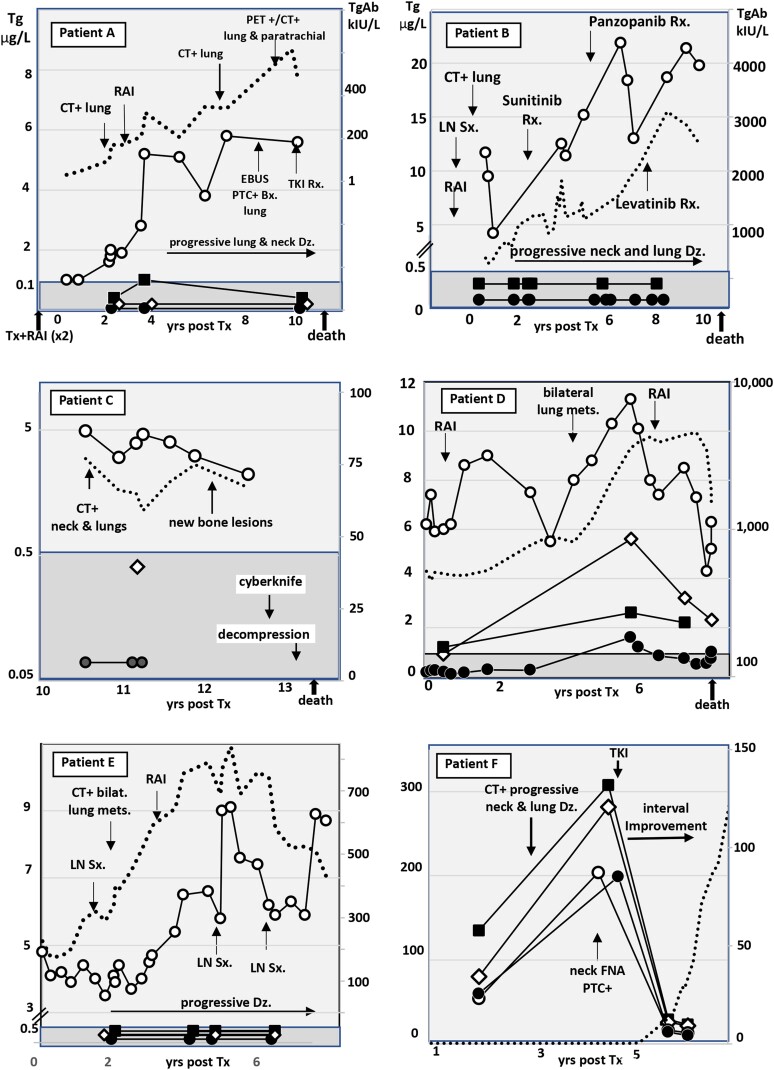
Serum Tg trends of 6 TgAb+ patients treated for progressive distant metastatic DTC in the years following initial treatment with thyroidectomy (Tx) and radioiodine (RAI). The serum Tg was measured by different methodologies: IMA (solid circles), MS-M (solid squares), MS-Q (open diamonds), and RIA (open circles). TgAb is shown by a dotted line.

Figure 2 shows Tg trends for 6 TgAb− DTC patients with progressive DMD measured in specimens drawn over a 1- to 12-year period. One patient (2C) died from DMD complications with a rising Tg trend prior to death that was observed with all methods. However, despite similar Tg trends, the between-method numeric Tg differences (mean 29%; range, 10%-148% CV) were as wide as for the entire TgAb− DTC patient group (Fig. 1A). Figure 3 shows Tg trends for 6 TgAb+ DTC patients with progressive DMD and stable or rising TgAb levels (dotted line), measured over a 1- to 10-year period with each methodology. Four patients died from DMD complications, 2 (A and B) with no Tg detected by either IMA or MS methodology. This paradoxical lack of Tg detection prompted confirmatory MS testing of fresh specimens drawn from each patient shortly prior to death. In contrast, Tg RIA measurements remained detectable and appeared to correspond appropriately for disease progression and therapeutic interventions. Patient F was notable in that following a de novo TgAb appearance shortly after initiating tyrosine kinase inhibitor (TKI) therapy, an approximate 20-fold fall in serum Tg was seen with all methods.

### Serum Tg in TgAb− and TgAb+ HY Patients

**Figure 4.**
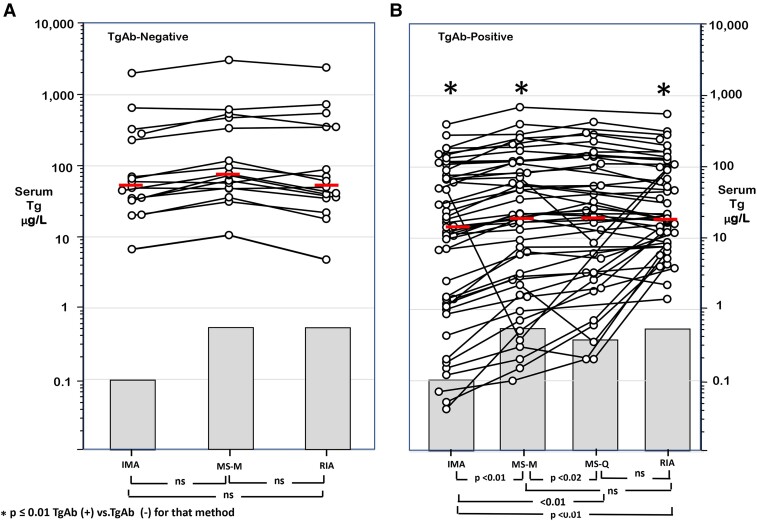
Serum Tg levels of hyperthyroid patients either without (panel a) or with (panel b) TgAb detected. Serum Tg was measured in the TgAb− patients using IMA, MS-M, and RIA and in TgAb+ patients by IMA, MS-M, MS-Q, and RIA. The horizontal bars indicate the medians, and the shaded areas indicate the detection limits of the methods.

TgAb− HY patients had comparable Tg levels reported by the different methods (median IMA 55 µg/L [range, 7-2012]; median MS-M 75 µg/L [range, 10-3060]; and median RIA 56 µg/L [range, 5-2400 µg/L]; all *P* > .10) (Fig. 4a).

The TgAb+ HY group had lower Tg levels measured by each method relative to their respective TgAb− group (median IMA 16 µg/L [range, < 0.1-395]; median MS-M 22 µg/L [range, < 0.2-691]; and median RIA 21 µg/L [range, 1.4 - 555 µg/L]) all *P* < .01. Tg IMA was lower (*P* < .01) than either MS-M, MS-Q, or RIA (Fig. 4b).

### TgAb in DTC vs HY Patients

**Figure 5.**
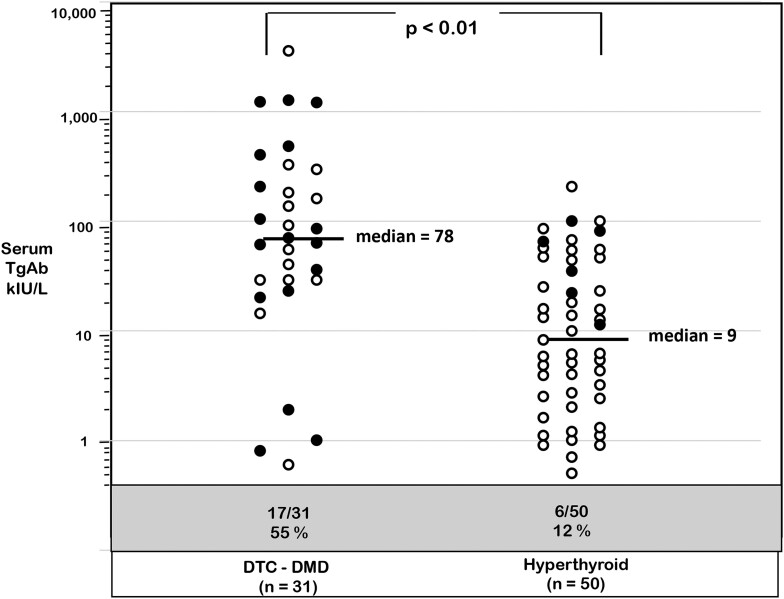
TgAb levels of the DTC vs HY patients. Solid symbols indicate patients who had no Tg detected by IMA and/or a MS test and are expressed as a percent in the shaded area.

As seen in Fig. 5, the DTC TgAb+ group had higher TgAb levels than the HY group (median 78 vs 9 kIU/L, *P* < .01). TgAb+ HY patients with no Tg detected by IMA or MS (solid symbols) had higher TgAb levels than those with Tg detected by IMA or MS (median TgAb 54 vs 6 kIU/L, *P* < .05). No such difference in TgAb levels was seen for DTC patients with no Tg detected by IMA and/or MS (median TgAb 63 vs 76 kIU/L, *P* < .10).

### TgAb/Tg Correlations in HY Patients

**Figure 6.**
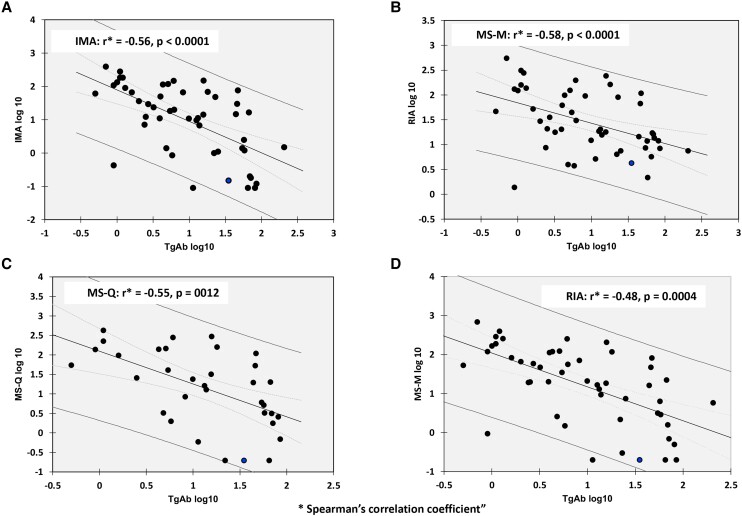
Regression analyses for log TgAb vs log Tg measurements made by the different methods on the TgAb+ HY patient group together with their respective Spearman correlation coefficients. Panel (a) IMA; (b) MS-M; (c) MS-Q; and (d) RIA.

As shown in Fig. 6, significant negative log correlations between the Tg and TgAb concentrations of TgAb+ HY patients were seen with all methods. Spearman correlation coefficients for the methods were: (a) IMA: *r* = −0.56, *P* < .0001; (b) MS-M: *r* = −0.58, *P* < .0001; (c) MS-Q: *r* = −0.55, *P* = .0012 and (d) RIA: *r* = −0.48, *P* = .0004.

## Discussion

This study evaluated the influence of TgAb on serum Tg measured by 3 different methodologies (IMA, MS, and RIA) in patients who were expected to have Tg detected as a result of either DTC with DMD, or due to hyperthyroidism (HY). When TgAb was absent, Tg was detected in all DTC and HY sera by all methods, albeit with between-method differences that exceeded the imprecision of the individual methods (∼30% vs ∼10%, respectively) (27, 29, 33). This suggested that both native (HY) and neoplastic (DTC) Tg is likely heterogeneous in nature (17, 27), necessitating method continuity when monitoring the Tg trends of DTC patients (3, 27). Despite these between-method differences, each method reported appropriate Tg trends and treatment responses for 6 TgAb− DTC patients with progressive DMD studied over a 1- to 12-year period. When TgAb was present, all methods reported lower Tg levels for both the DTC and HY groups, relative to their respective TgAb− group. This suggested that the presence of TgAb reduces Tg measured by all methodologies—a TgAb-lowering effect that was supported by the inverse log TgAb/Tg relationships seen with all methods for the TgAb + HY patient group (Fig. 6), and the rapid Tg decline following a de novo TgAb appearance in a DTC patient with progressive DMD (Fig. 3F). This study confirms other reports (6, 33, 38-40) that 20% to 50% of TgAb+ patients with structural DTC have no Tg detected by either IMA or MS, making the practice of reflexing TgAb+ specimens to MS, with the goal of avoiding TgAb effects, an ineffective strategy. Although the RIA detected Tg in most (> 90%) TgAb+ DTC patients, and reported appropriate Tg trends and treatment responses for TgAb+ patients with progressive disease (Fig. 3), TgAb also appeared to lower RIA measurements as evident from the lower Tg RIA of both the TgAb+ DTC and HY groups vs their respective TgAb− group (DTC: 4 vs 36 µg/L, *P* < .01 and HY: 20 vs 56 µg/L, *P* < .01) and the negative log TgAb/Tg RIA relationship shown in Fig. 6D. It was surprising that 5% of TgAb+ HY patients had no Tg detected by IMA or MS and an additional 25% had paradoxically low (< 3 µg/L) Tg IMA and MS tests despite clinical and biochemical hyperthyroidism. This suggested that TgAb had a lowering effect on both neoplastic and native Tg measured by all methodologies, possibly reflecting a TgAb-mediated increase in Tg metabolic clearance (50-52). The TgAb-lowering effect found by this study may compromise the use of Tg as a tumor marker for TgAb+ DTC patients. Some guidelines now suggest that sensitive TgAb detection, and the monitoring of the TgAb trend as a surrogate DTC tumor marker can be a useful clinical parameter to monitor (3, 4, 42, 43).

Prior Tg MS studies of DTC patients with structural disease have either not specified the site of disease or included patients with both local (cervical lymph node) and distant metastatic disease (6, 33, 39, 40). This investigation focused on DTC patients with DMD because cervical lymph nodes may not secrete sufficient Tg to be detected in serum (53). The current study found that ∼50% of TgAb+ DTC patients had no Tg detected by IMA or MS—a percentage comparable to a recent study (40) but higher than our previous study (33), lending support to the practice of following the TgAb trend as a surrogate tumor marker (3, 4, 42, 43) or remeasuring Tg by RIA methodology. It should be noted that the current DTC/DMD patient group had 10-fold higher TgAb levels than our previous cohort (33), consistent with the higher disease burden (DMD) of the current patient group (54). Given the negative TgAb/Tg relationships shown for all methodologies (Fig. 6), and the rapid Tg decline following a de novo TgAb appearance in the DTC patient shown in Fig. 3F, patients with a high TgAb might be expected to have a lower Tg measured by all methodologies.

Whether the TgAb-associated Tg-lowering is an in vivo or in vitro effect remains undetermined. There could be TgAb-mediated accelerated clearance of Tg-TgAb complexes (Tg is absent) (7, 50-52). Alternatively, TgAb could interfere with the detection of Tg in the specimen (Tg is present). Mechanisms for TgAb interference could be multifactorial and relate to either Tg and/or TgAb heterogeneity (29, 35, 55-61). Immunoassay methodologies (IMA and RIA) use antibody reagents to bind conformational epitopes on the quaternary Tg protein structure (62, 63), whereas the MAbs employed by IMAs have limited epitope targets (1, 36) that are more prone to TgAb masking than the polyclonal antibodies used for RIA (10, 62). In contrast, Tg MS detection relies on the generation of a tryptic Tg peptide that can be immunocaptured and quantified by LC-MS/MS (64). Failure to detect Tg using MS could result from gene polymorphisms giving rise to conformationally abnormal neoplastic Tg proteins (2, 29), abnormal glycosylation (21, 65), and/or structural abnormalities resulting from the low iodine content typical of neoplastic Tg (24, 25, 30). It should be noted that Tg detection was essentially the same using 2 different MS methods (MS-M and MS-Q) that targeted different Tg peptides. This suggested that the Tg detected was conformationally similar, a contention supported by showing appropriate Tg measurements for FNAW specimens (TgAb−) from 3 TgAb+ DTC patients with no serum Tg detected by IMA or MS. These observations suggested that the presence of TgAb itself, rather than Tg molecular heterogeneity, was likely responsible for lower Tg measurements in the presence of TgAb, even with MS methodology.

Although TgAb appeared to have less effect on the RIA vs IMA or MS Tg, a TgAb-lowering effect on the RIA Tg levels was evident from: (i) the negative TgAb/Tg RIA relationship shown in in Fig. 6D; (ii) the paradoxical absence of Tg detected by RIA in 2 TgAb+ DTC patients; (iii) lower Tg RIA values for both the TgAb+ DTC and HY groups relative to their respective TgAb− group; and (iv) the dramatic fall in RIA seen after the de novo TgAb appearance seen in Fig. 3F. However, despite these TgAb effects, a rising Tg RIA trend was seen for patients with progressive DMD (Fig. 3) concomitant with a rising TgAb trend—the latter increasingly being recognized as a surrogate DTC tumor marker (3, 4, 42, 43). Few Tg RIA methods remain clinically available, and existing methods differ in clinical performance resulting from employing different reagents (6, 37, 66). Thus, until the clinical sensitivity of MS methodology can be improved, the TgAb trend (measured with the same method) may remain the most reliable (albeit surrogate) DTC tumor marker for monitoring TgAb+ DTC patients (3, 4, 42, 43). Claims that MS methodology is free from TgAb effects remain controversial. Such claims were based on finding that TgAb+ sera had higher Tg MS than Tg IMA (15, 30), “appropriate” recoveries of native Tg added to TgAb+ sera (14), and MS vs RIA correlations in TgAb+ sera (14). However, subsequent clinical studies have reported that a high percentage of TgAb+ DTC patients with structural disease have no Tg detected by MS, suggesting that TgAb can influence the clinical utility of Tg MS methodology (6, 33, 38-40).

The current study found that TgAb appears to have a lowering effect on both neoplastic Tg (DTC) and native Tg (hyperthyroidism) measured by all 3 methodologies (IMA, MS, and RIA). This suggests that the most reliable strategy for monitoring TgAb+ patients for persistent/recurrent DTC would be anatomical imaging used in conjunction with monitoring the TgAb trend (3, 4, 42, 43), provided that TgAb is measured by the same method in preferably the same laboratory (3, 44).

## Data Availability

Restrictions apply to the availability of some or all data generated or analyzed during this study to preserve patient confidentiality or because they were used under license. The corresponding author will on request detail the restrictions and any conditions under which access to some data may be provided.

## Abbreviations

DTC: differentiated thyroid cancer
FNA: fine needle aspiration
FNAW: fine needle aspiration washout
FT3: free triiodothyronine
FT4: free thyroxine
HY: hyperthyroidism
IMA: immunometric assay
LC-MS/MS: liquid chromatography–tandem mass spectrometry
Mab: monoclonal antibody
MS: mass spectrometry
MS-M: Mayo Medical Laboratories MS test
MS-Q: Quest MS test
RIA: radioimmunoassay
Tg: thyroglobulin
TgAb: Tg autoantibody
TSH: thyrotropin (thyroid stimulating hormone)
